# Effect of high-intensity exercise training on functional recovery after spinal cord injury

**DOI:** 10.3389/fneur.2025.1442004

**Published:** 2025-02-17

**Authors:** Xinyan Li, Qianxi Li, Chenyu Li, Chunjia Zhang, Jinghua Qian, Xin Zhang

**Affiliations:** ^1^School of Sports Medicine and Rehabilitation, Beijing Sport University, Beijing, China; ^2^School of Rehabilitation, Capital Medical University, Beijing, China

**Keywords:** athletic training, spinal cord injury, exercise intensity, high-intensity training, functional recovery

## Abstract

Spinal cord injury (SCI) is a severe disorder of the central nervous system characterized by high prevalence and significant disability, imposing a substantial burden on patients and their families. In recent years, exercise training has gained prominence in the treatment of SCI due to its advantages, including low cost, high safety, ease of implementation, and significant efficacy. However, a consensus regarding the effects of various exercise training modalities and intensities on functional recovery in individuals with SCI remains elusive, and the efficacy and risks associated with high-intensity exercise training (HIET) are subjects of ongoing debate. Some studies have indicated that HIET offers superior therapeutic benefits, such as enhanced cardiovascular stress reflex sensitivity and increased release of neurotrophic factors, compared to moderate- or low-intensity exercise training. Nonetheless, HIET may entail risks, including secondary injuries, heightened inflammatory responses, and falls. This study reviews the positive and negative effects of HIET on various body systems in individuals with SCI, focusing on mechanisms such as neuroplasticity and immune regulation, to provide a theoretical basis and evidence for its prospective clinical application. Additionally, the limitations of existing studies are analyzed to inform recommendations and guidance for future research.

## 1 Introduction

Spinal cord injury (SCI) is a prevalent, highly disabling, and progressive neurological condition (1). Currently, more than 20 million people worldwide suffer from SCI, and from 1990 to 2019, the prevalence of SCI increased by 81.5%, incidence by 52.7%, and years lived with disability (YLDs) by 65.4% (2). SCI can be categorized into primary and secondary injuries. Primary injuries typically involve axonal damage, vascular disruption, and cellular membrane destruction, while secondary injuries comprise a cascade of responses to primary trauma, including inflammation, ischemia, vascular dysfunction, free radical formation, apoptosis, and necrosis (3). Current treatment modalities for SCI are predominantly invasive and include surgical decompression, neural bridging, neurostimulation and neuromodulation, brain-computer interfaces, and stem cell therapy (4). By contrast, exercise training represents a cost-effective and non-invasive treatment option with fewer adverse effects (5–7). Exercise training is increasingly employed as a comprehensive treatment approach that integrates multiple therapeutic strategies (8).

Exercise training has been reported to achieve efficacy comparable to pharmacotherapy (9), irrespective of the level of injury (10, 11). It leverages residual muscle strength to activate the remaining musculature (12) and provides benefits such as fat reduction, muscle development, metabolic enhancement, blood pressure regulation, and increased bone density (13, 14) (see Table 1 [1, 2]). These improvements contribute to enhanced functional independence, mental health, and quality of life for patients (12, 15). Importantly, individuals with SCI must maintain a high level of exercise intensity to achieve functional improvements (16) (see Table 1 [3]). Extremely low-intensity exercise may yield limited benefits compared to high-intensity exercise training (HIET) (16–18) (see Table 1 [3, 4]). Studies have indicated that HIET with lower total training volume induces greater physiological adaptations than moderate-intensity exercise (19). However, the current clinical use of HIET remains conservative due to safety concerns, and lack of consensus on HIET’s efficacy and risks.

**Table 1:** Clinical trials of HIET after SCI.

Reference	Study design	Subjects	HIET program	Assessments	Outcome
[1] United States (13)	RCT; Hybrid functional electrical stimulation rowing	(1) 60 individuals with SCI, aged 18–40; (2) AIS = A–C, NLI=C1-T10; (3) All patients were 3–24 months post-injury and had been discharged from inpatient rehabilitation to the community prior to enrollment.	Mixed-function electrically stimulated rowing for whole-body exercise for 30–60 min, 2–3 times per week, 6 months, with a target heart rate > 75% HR_max_.	Cardiovascular stress reflex sensitivity assessed by neck aspiration technique every 3 months.	In patients with SCI, 6 months of high-intensity whole-body exercise and FES significantly improved cardiovascular stress reflex sensitivity.
[2] United States (14)	RCT; HIET via the addition of FES	(1) 31 individuals with SCI, aged 18–40; (2) AIS = A-C, NLI=C5-T12; (3) BMI within normal to overweight range; (4) Wheelchair users.	The maximal FES rowing test; 70–85% of VO_2peak_ for 30–40 min, 3 times/week.	Exercise capacity; Dual x-ray absorptiometry; Insulin sensitivity and cardiovascular health markers; Basal metabolic rate.	FESRT early after spinal cord injury provides sufficient stimulation to attenuate deleterious body composition changes. This may lead to prevention of loss of lean mass, including bone.
[3] Brazil (16)	NRCT; Treadmill	(1) 19 wheelchair-bound individuals with SCI, 12 AB controls, aged ≥18 years; (2) Duration of disease≥1 year; (3) NLI = T7-L1; (3) Complete traumatic SCI.	Participants underwent three exercise sessions in treadmills at different relative intensities: at VT1 intensity, 15% below VT1, and 15% above VT1. HIET were designed to achieve the speed that corresponds to VO2 at 15% above VT1.	Energy expenditure; Respiratory variables; IL-1ra and IL-1β concentrations were assessed by commercial ELISA; IL-2, IL-4, IL-6, IL-10 and TNF-α concentrations were assessed by MULTIPLEX assay.	Persons with SCI may need to engage in higher volume or energy-expending physical activity than able-bodied to achieve anti-inflammatory effects similar to those of acute exercise.
[4] United Kingdom (18)	Cohort study; Handcycle, Arm Crank Ergometry, Wheelchair	(1) 134 individuals (males: 98; females: 36); (2) Participants were split into those with paraplegia (PARA), tetraplegia (TETRA), or alternate health condition (Non-SCI); (3) Competitive athletes, competing at a national or international level.	A submaximal step test with 3 min stages. HC and ACE tests start at 15–60 W and increase by 10–20 watts every 3 min; WCP tests start at 0.7–2.8 m/s and increase by 0.2–0.4 m/s every 3 min.	Heart rate and VO_2_ were monitored throughout, and capillary blood samples were collected from the earlobes at the end of each phase to measure the lactate threshold.	Aerobic exercise intensity prescriptions for adults with SCI should not be based on fixed %V̇O2_peak_ and %HR_peak_, as this method does not allow for an even distribution of exercise intensity domains.
[5] United States (23)	RCT; Arm crank exercise	(1) 27 individuals with SCI (14 females, 13 males), aged 18–65; (2) NLI = T2-L5; (3) Duration of disease 1 years; (4) Self-reported use of a wheelchair 75% of waking days, weight stabilization (no change in weight ≥ 3%).	Perform 30 min of arm cranking (60s intervals, 80–90% peak heart rate) 4 times per week for 6 weeks.	Fasting insulin; PPO; VO_2peak_.	A 6-week HIIT intervention improved upper extremity peak power output and postprandial insulin sensitivity. There were no other beneficial effects on a wide range of cardiometabolic component risk factors.
[6] United States (26)	RCT; Treadmill	(1) 19 individuals with SCI, aged 18–75; (2) NLI > T10; (3) Duration of disease 6; (4) Ability to independently complete at least three speeds on a graded intensity treadmill test.	Started walking at 0.1 m/s and increased speed by 0.1 m/s every 2 min, 100% maximum speed until the subject requires support from the seat belt or voluntarily stops the test.	Concentrations of BDNF; IGF-1; Measures of cardiorespiratory dynamics.	Persons of incomplete SCI single exercise-dependent changes in peripheral BDNF are related to the relative intensity of exercise movements, high-intensity exercise may promote changes in neuroplasticity, and intensity may be an important parameter for physical rehabilitation interventions after neurologic injury.
[7] China (47)	RCT; MOTOmed Intelligent Exercise Trainer	(1) 60 individuals with SCI, aged 18–65; (2) AIS=B-D, NLI=C4-L2; (3) Duration of disease 2–12 months, Ashworth = I ~ III, Tardieu = 1–5; (4) Not using an antitussive drug, or taking a stable type or dose of that drug for more than 1 month.	MOTOmed intelligent exercise trainer; BPE = 14–15; 30 min each time., once a day, 5 d/week for 4 weeks.	The degree of spasticity was assessed before and after 4 weeks of treatment, and serum BDNF concentrations were analyzed before and after the patients’ treatment.	Exercise training, especially HIET, helps to improve spasticity in the lower limbs of patients with incomplete SCI and increase serum BDNF levels, and there is a positive correlation between the intensity of exercise training, the degree of improvement in spasticity, and the growth rate of serum BDNF levels.
[8] United States (52)	Crossover design; Treadmill	(1) Aged 18–75; (2) AIS=C-D, NLI ≥ T10; (3) Duration of disease ≥1 year; (4) Demonstrates intact quadriceps or plantar flexor tendon reflexes; (5) Ability to walk on the ground without physical assistance at a walking speed of <1.0 m/s, with the use of assistive devices (e.g., walkers or canes) and below-knee braces as needed.	70–85% of predicted HR_max_ rate at this age for 4–6 weeks of running table exercise for 20 h.	Spatiotemporal variables, sagittal-plane gait kinematics, and neuromuscular synergies from electromyographic (EMG) recordings.	Further improvements in neuromuscular coordination were primarily found after HIET, although their contribution to improved motor performance (i.e., speed) is unclear.
[9] United States (57)	RCT; Arm Crank Force Gauge	(1) 7 individuals with SCI (6 males, 1 female), aged 51.3 ± 10.5; (2) AIS = A-D, NLI=C5-L2; (3) Duration of disease 3 years.	Arm cranking exercise, 30s × four repetitions; rest 4 min, two times per week, 50% peak power: 145 ± 62 W; 25% HRR: 15 ± 1.2 W; 6 weeks.	Aerobic capacity, muscle strength, lipids, glucose tolerance, blood pressure and body composition.	There was no difference between MIT and HIIT. Both conditions led to improvements in insulin sensitivity, aerobic capacity, muscle strength, and lipids in patients with spinal cord injury. Future larger cohort studies are needed to determine whether the shorter duration required for HIIT is preferable to current exercise recommendations for MIT.
[10] Canada (58)	Randomized trial; Arm-Crank Dynamometers	(1) Aged 18–65; (2) Duration of disease<365 days; (3) NLI ≤ C2.	3 × 20-s “all-out” cycle sprints (≥100% peak power output) interspersed with 2 min of active recovery (10% peak power output; total commitment time, 10 min), three times per week for 5 weeks.	Peak power output; Submaximal arm-crank ergometry performance; exercise satisfaction, exercise self-efficacy and pain.	In subacute persons with SCI, 5 weeks of SIT treatment improved physical abilities to the same extent as MICT, despite the significantly shorter duration of SIT.
[11] United States (68)	Randomized crossover; Wallmounted electronically braked arm crank ergometer	(1) 10 adult males with SCI; (2) AIS = A-C, NLI ≤ T1.	All exercises were performed on a wall-mounted electronic braked arm crank ergometer with 2 min of rest for every 2 min of exercise at an intensity >80% of VO_2max_, and this was repeated 3 times.	Apply appropriate stoichiometric equations to indirectly analyze calorimetric data by detecting exhaled gas composition and content.	Compared to MICE, HIIE imposes a greater physiological stimulus while requiring a shorter period of time to achieve the target caloric expenditure. Therefore, exercise intensity may be an important consideration in adapting exercise prescriptions to address cardiometabolic comorbidities of spinal cord injury.

SCI, spinal cord injury; HIET, high-intensity exercise training; HIIT, high-intensity interval training; MICE, moderate-intensity continuous training; MIT, moderate intensity training; SIT, sprint interval training; AIS, American Spinal Cord Injury Society Injury Scale; HR_peak_, peak heart rate; HR_max_, maximum heart rate; HRR, heart rate recovery; NLI, Neurologic Level of Injury; RCT, randomized controlled trial; NRCT, non- randomized controlled trial; VT1, ventilatory threshold 1; VO_2_, oxygen consumption; VO_2peak_, peak aerobic capacity; VO_2max_, maximal oxygen uptake; PPO, peak power output; BDNF, brain-derived neurotrophic factor; IGF-1: Insulin-like growth factor-1; BPE, Perceived Exertion Scale.

Currently, there are no standardized criteria for exercise intensity in individuals with SCI. Most clinical studies have assessed exercise intensity based on heart rate or speed. This review included studies in which HIET was explicitly implemented for subjects with SCI, with exercise intensity defined through heart rate ranges, exercise loads, or similar parameters. Based on the literature, HIET is defined as 75–100% of the maximum heart rate or 70–90% of the maximum speed, adjusted for individual differences. In animal studies, HIET criteria often include 70–85% of maximum walking speed or self-defined greater walking speed and 80–85% of maximum heart rate. Further research is necessary to optimize these criteria and develop effective exercise training protocols to facilitate recovery in individuals with SCI.

The potential benefits and applications of high-intensity interval training (HIIT), a specific form of HIET involving repetitive high-intensity workouts with short rest intervals, have been detailed in existing literature. However, comprehensive reviews of other types of HIET, including animal experiments, remain scarce (20). This review focuses on the effects of HIET, encompassing HIIT and other high-intensity exercise modalities, on functional recovery after SCI. It examines exercise protocols in current studies, highlights relevant shortcomings, and provides recommendations while elucidating the advantages of HIET.

## 2 Positive effects of HIET on functional recovery after SCI

### 2.1 Cardiopulmonary benefits

HIET has been shown to significantly enhance postprandial insulin sensitivity, blood pressure regulation, maximal oxygen consumption, and systemic vascular function, thereby exerting positive effects on the cardiovascular and pulmonary systems.

SCI often results in impaired respiratory muscle function, cardiorespiratory dysfunction, and diminished aerobic capacity, which collectively reduce cardiopulmonary reserve and increase the risk of cardiovascular diseases (21). Compared to low-intensity exercise, HIET improves postprandial insulin sensitivity, thereby lowering obesity rates and cardiac burden, although it does not significantly affect a wide range of cardiometabolic risk factors (22, 23) (see Table 1 [5]). Additionally, autonomic dysreflexia, a condition frequently associated with SCI, can cause abnormal blood pressure fluctuations, underscoring the importance of blood pressure regulation for cardiovascular health. One study (13) (see Table 1 [1]) demonstrated that HIET enhances cardiovascular stress sensitivity compared to low-intensity exercise training under similar conditions. However, a single HIET session may not significantly improve stress sensitivity. To achieve meaningful cardiovascular benefits, individuals with SCI may require high-intensity whole-body exercise combined with mixed-function electrical stimulation. Notably, HIET has been associated with significant improvements in maximal oxygen consumption and systemic vascular function compared to low-intensity exercise training (24).

### 2.2 Neurological benefits

#### 2.2.1 Spinal nerves

**Figure 1:**
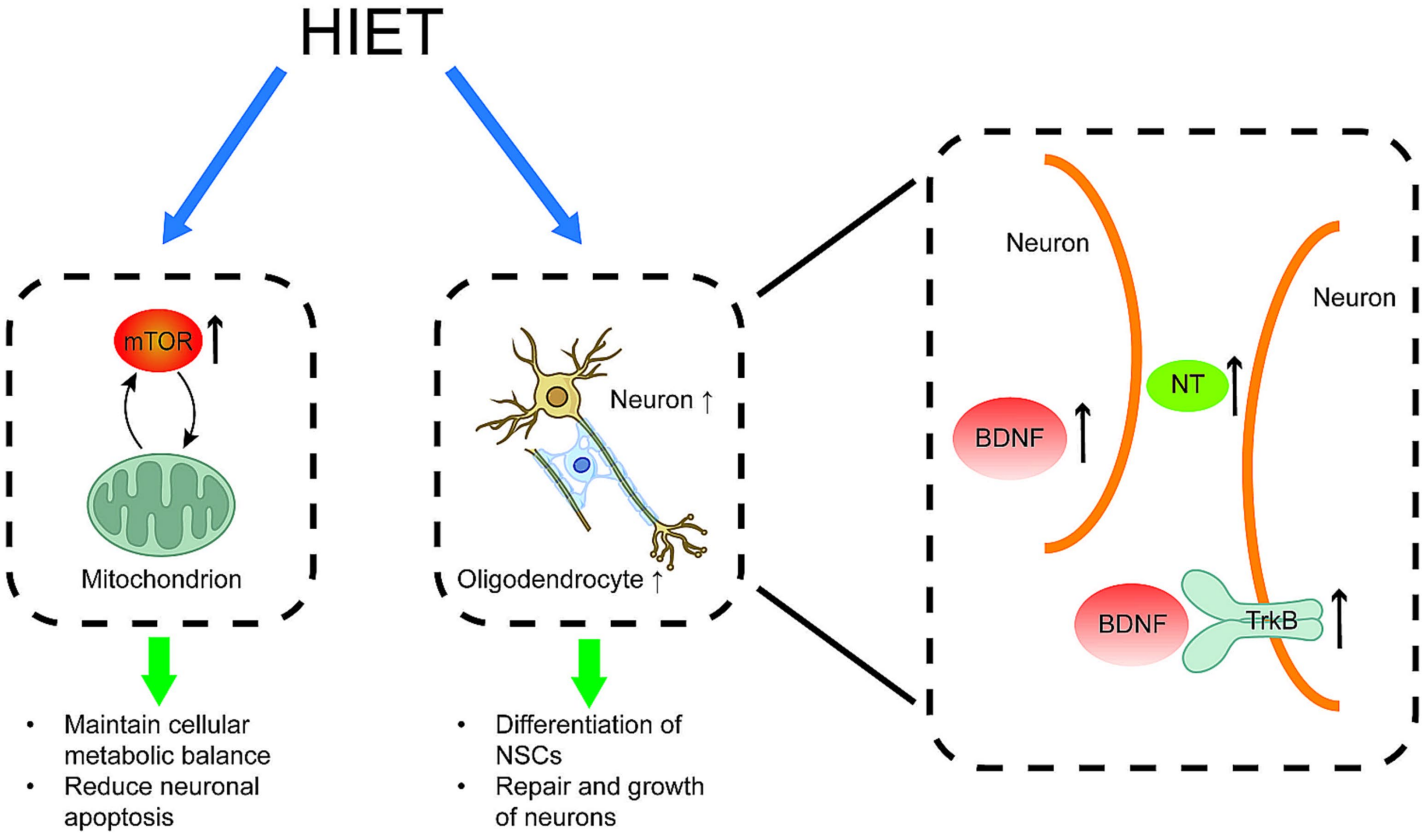
Positive effects of HIET on spinal cord histopathological changes HIET has been shown to elevate the levels of BDNF, mTOR, TrkB proteins, and neurotrophic factors (NT) in spinal cord tissues. These changes promote the formation of oligodendrocytes, the differentiation of neural stem cells (NSCs), protection of mitochondria and the repair and growth of normal spinal cord neurons. BDNF: brain-derived neurotrophic factor; mTOR: mammalian target of rapamycin; NT: neurotrophic factor; TrkB protein: tyrosine kinase receptor B protein; NSCs: neural stem cells.

HIET upregulates the expression of brain-derived neurotrophic factor (BDNF) and the mammalian target of rapamycin (mTOR) in the spinal cord tissues of rats with SCI. This activation of the mTOR pathway protects mitochondrial quantity and quality, inhibits neuroglial cell activation, and promotes the repair of spinal cord nerves (Figure 1).

Exercise training enhances neuroplasticity by promoting myelin structural repair, neurotrophin (NT) secretion, and the proliferation and differentiation of endogenous neural stem cells (NSCs) (25). However, these molecular mechanisms require a specific level of exercise intensity, particularly NT, which is strongly dependent on exercise intensity (26) (see Table 1 [6]). BDNF facilitates the differentiation and maturation of oligodendrocytes, enhancing spinal cord neuroplasticity and promoting neural pathway repair (27). Studies (28, 29) (see Table 2 [1, 2]) have demonstrated that high-intensity weight-loss walking training in SCI rat models significantly promotes the synthesis and transport of endogenous pro-myosin receptor B (TrkB) and BDNF and increases the number of Nysted’s vesicles in spinal cord tissues. In contrast, low-intensity training did not produce similar effects, failing to elevate TrkB and BDNF levels despite upregulating BDNF expression compared to non-exercising rats.

**Table 2:** Animal experiments of HIET after SCI.

References	Subjects	HIET program	Assessments	Outcome
[1] China (28)	*n* = 50, T10 vertebral spinal cord contusion in rats	BWSTT was initiated 14 days after SCI, 15 min/repetition, 1 repetition/day, 5 days/week, with a training cycle of 3 weeks. HIET was performed at a walking speed of 21 cm/s.	Hip, knee, and ankle walking, trunk movement, and coordination; TrkB and BDNF protein expression levels in spinal cord tissue; Spinal cord tissue morphology.	The use of medium- and high-intensity BWSTT after SCI can significantly improve the limb motor function of patients, and its mechanism of action may be related to the increase in the level of TrkB and BDNF protein expression and the number of Nysted’s vesicles in the spinal cord, which improves the morphology and the number of spinal cord neuronal cells and protects the damaged neuronal cells.
[2] China (29)	*n* = 10, C5 SCI mice	BWSTT, HIET set at 70% of maximal exercise speed. Injury rest for 1 week, followed by formal treadmill training for 30 min/repetition, 1 repetition/day, 5 days/week for 4 weeks.	Neurophysiological tests; Western blotting; immunofluorescence NeuN and p-S6 co-staining; Golgi staining; BDA tracing.	First, no significant increase in cortical neurotrophic factor expression and activation of the mTOR pathway was observed in the LEI group compared to the MEI and HEI groups, thus selecting higher than low exercise intensities is more beneficial to SCI recovery from a comprehensive and long-term perspective.
[3] China (33)	*n* = 20, 8-month-old male APP/PS1 transgenic mice; *n* = 10, C57BL/6 wild-type mice	BWSTT, running fast at 85% VO_max_ (5.25 m/min) intensity for 3 min, intermittent slow running at 40% VO_max_ (5.15 m/min) intensity for 1 min, repeated for 10 cycles, total exercise time 40 min, five times/week for 12 weeks.	Animal learning and memory capacity; mitochondrial membrane potential; rate of mitochondrial reactive oxygen species production; expression of brain hippocampus-associated proteins.	HIIT may improve mitochondrial function and reduce hippocampal Aβ accumulation in APP/PS1 transgenic AD mice by mountain white stripe BDNF, which in turn activates AMPK-PINK1/Pakin-mediated mitochondrial autophagy, and improves memory and learning ability.
[4] China (48)	*n* = 40, Male SD rats without any treatment	BWSTT, HIET group with primary loading intensity of 80% of the maximal oxygen uptake, 5 days/week, 60 min/day, 12 weeks.	Bone density; Bone structure; Bone metabolism; Completion of maximal oxygen uptake testing exercise intensity.	Endurance exercise interventions of different intensities improved bone mineral density, bone structure, bone tissue, and serum indices to different degrees in growing rats. HIET was the most effective in improving bone mineral density, bone structure, bone organization and serum indexes in growing rats, followed by medium-intensity endurance exercise, and finally small-intensity endurance exercise.
[5] China (51)	*n* = 30, T10 vertebral SCI in SPF-grade adult female SD rats using the modified Allen’s impingement method	BWSTT, the speed of the running platform was set at 6 meters/min, and each training session lasted 20 min, 2 times a day (with an interval of not less than 2 h), 5 days a week for a total of 4 weeks of training.	Motor function of the hind limb; degree of calf triceps spasticity in the hind limb; neurophysiologic detection of the -H reflex; immunohistochemical techniques; protein immunoblotting.	Exercise training can promote the recovery of motor function and effectively relieve spasticity in SCI rats; its spasticity-relieving effect is related to the exercise-induced increase in BDNF/TrkB synthesis, increase in the expression of pCREB and the activation of CREB, and the promotion of the expression of GAD65, GAD67, GABAB receptor and KCC2.
[6] China (62)	*n* = 60, C5 crush SCI in female mice	BWSTT, running table exercise at 70% of the maximum speed recorded in the pre-experimental test, 4 weeks, 30 min/days, 5 days/week	Recovery of motor function; cortical mechanism target proteins of mTOR pathway-related proteins; activation of the mTOR pathway and axon germination; and changes in neuronal plasticity in the motor cortex.	The expression of neurotrophic factors in the motor cortex and the activation of the mTOR pathway depend on appropriate exercise intensity, while excessive exercise intensity leads to negative effects.
[7] China (65)	*n* = 80, SD male rats	BWSTT, running table speed 26.8 m/min, running table inclination 10°, 85% VO_2max_, days at the same time and place at the same exercise intensity training for 1 h, 14 days.	Changes in body weight; alterations in skeletal muscle micro- and ultrastructure; and expression of the phase of skeletal muscle HIF-la mRNA.	Repeated high-intensity exercise causes weight loss, disorganization of myofilament arrangement, degeneration and necrosis of some myocytes, mitochondrial swelling, and mechanism of skeletal muscle HIF-1a mRNA expression in rats.
[8] China (66)	*n* = 24, T10 vertebral spinal cord contusion mice	BWSTT, HIET at 21 cm/s, starting 14 days after SCI for 3 weeks, 5 days/week, 1time/day, 15 min per time(no intervals in between).	Number of bud axon intersections; nerve cell protrusion intersections; behavioral manifestations.	Compared with the MEI and HEI groups, no significant increase in cortical neurotrophic factor expression and activation of the mTOR pathway were observed in the LEI group, thus selecting exercise training at higher than low exercise intensities is more beneficial to the recovery of spinal cord injury patients from a comprehensive and long-term perspective.
[9] China (50)	*n* = 45, Male SD rats with body mass of 150–170 g	BWSTT, at a speed of 28 m/min 4 times/day for 3 days for 10 min each time, interspersed with 10 min of rest.	Plasma creatine kinase; superoxide dismutase levels; apoptosis in gastrocnemius muscle cells; AMPK phosphorylation levels in gastrocnemius muscle tissue; GLUT4 expression and translocation in gastrocnemius muscle tissue.	Sprint interval exercise significantly attenuates skeletal muscle cell injury induced by exhaustion exercise; sprint interval exercise induces adaptive changes in AMPK, improves the level and efficiency of AMPK phosphorylation during subsequent exhaustion exercise, and enhances the duration of exhaustion exercise in rats by promoting the expression and translocation of GLUT4 in skeletal muscle.

SCI, spinal cord injury; HIET, high-intensity exercise training; HIIT, high-intensity interval training; BWSTT, Body Weight Support Treadmill Training; BDNF, brain-derived neurotrophic factor; TrkB, Tropomyosin receptor kinase B; mTOR, mammalian target of rapamycin; AMPK, Adenosine 5′-monophosphate (AMP)-activated protein kinase; AD, Alzheimer’s disease; LEI, Low exercise intensity group; MEI, Medium exercise intensity group; HEI, High exercise intensity group.

The mTOR pathway plays a pivotal role in exercise-induced nerve growth. Zhan (29) (see Table 2 [2]) found that mTOR expression significantly increased in spinal cord tissues of SCI rats following HIET, triggering endogenous axonal regeneration. Mitochondria, vital for cellular metabolism, produce ATP molecules via phosphorylation. Neurons require adequate energy for survival; mitochondrial dysfunction leads to neuronal apoptosis (30). Post-SCI, altered mitochondrial morphology and function, including Ca^2+^ disorders, initiate cascade reactions leading to neuronal death (31).

Few studies have assessed the effects of HIET on mitochondria post-SCI. Research on neurodegenerative diseases (32, 33) (see Table 2 [2, 3]) indicates that HIIT preserves mitochondrial quantity and quality to meet neuronal energy demands. This preservation occurs through BDNF-mediated activation of the AMPK/PINK1/Parkin pathway in Alzheimer’s disease models. Furthermore, HIIT enhances mitochondrial membrane potential, reduces reactive oxygen species (ROS) production, and decreases amyloid-*β* peptide levels in the hippocampus. HIIT also exhibits anti-inflammatory effects by inhibiting glial cell activation and reducing inflammatory cytokine release, protecting neurons from damage and preventing apoptosis. Additionally, HIIT increases lactate levels, which regulate mitochondrial quality and promote BDNF expression (34). However, further studies are necessary to confirm whether HIIT affects spinal cord neuronal mitochondria after SCI.

#### 2.2.2 Brain neurons

SCI-induced denervation triggers apoptosis and atrophy of brain neurons, resulting in the loss of afferent information in somatosensory brain regions and impaired motor innervation throughout the body. Consequently, the sensory-motor cortex undergoes extensive reorganization of neuronal circuits, altering the electrical activity of neural populations in affected regions (35, 36). SCI can also cause cognitive deficits, potentially due to chronic inflammation and glial activation. Elevated pro-inflammatory factors in the brain after SCI hinder neurogenesis and lead to neurodegeneration (37–39).

HIIT ameliorates cerebral neurodegeneration by upregulating hippocampal PINK1, Parkin, and BDNF proteins, promoting AMP-dependent protein kinase expression, and reducing amyloid-*β* protein accumulation in Alzheimer’s disease models. These effects improve memory and learning abilities (33) (see Table 2 [3]). Studies on exercise training in SCI models show increased IL-6 levels and reduced pro-inflammatory cytokines, such as IL-1β and TNF-*α*, in the hippocampus. Exercise also decreases IFN-*γ* levels, counteracting chronic brain inflammation. Additionally, exercise promotes selective transport of the synaptic protein SNAP25, induces PGC-1α and SIRT1 upregulation, reduces p53 acetylation, and increases mitochondrial respiratory complex content, thereby regulating brain plasticity and activating neuroprotective pathways (40). Nevertheless, further research is needed to elucidate the effects of HIET on the brain microenvironment and on neuronal remodeling and repair.

#### 2.2.3 Peripheral nerves

SCI often extends to remote regions, inducing secondary plastic changes in the peripheral nervous system. It disrupts motor signal transmission, resulting in prolonged limb immobility, secondary complications from compression or inactivity, and potential atrophy and degeneration of motor neuron pools distal to the lesion (41). Studies suggest that intermittent exercise of any intensity can promote axonal growth in injured peripheral nerves, with HIIT showing more pronounced effects. The intensity of exercise is directly proportional to neurotrophic factor content, which enhances the proliferative activity of peripheral neuron precursor cells. This activity promotes neuronal migration to injured areas, mitigates apoptosis, and stimulates growth of movement-related axons, facilitating peripheral nerve repair (41, 42).

### 2.3 Immune benefits

HIET can modulate the inflammatory response by balancing pro-inflammatory and anti-inflammatory factors. The immune system primarily drives the inflammatory response in spinal cord tissues following SCI, which induces a neuroinflammatory reaction predominantly mediated by microglia (MG) and macrophages within the tissues (43). Subsequently, platelets release cytokines, chemokines, and eicosanoids, initiating neutrophil infiltration. Activated MGs secrete significant quantities of pro-inflammatory factors, resulting in extensive infiltration of inflammatory cells and cytokines and delaying leukocyte recovery (43, 44).

Regulatory T cells (Tregs) represent a subset of T cells that regulate autoimmune reactivity *in vivo* and play an anti-inflammatory role following SCI. Walsh et al. (45) reported that HIET increases Treg levels more effectively than low- and moderate-intensity training, thereby suppressing the inflammatory response in spinal cord tissues and mitigating the secondary damage caused by excessive inflammation. Another study (16) (see Table 1 [3]) involved both SCI patients and able-bodied individuals undergoing three exercise sessions at varying relative intensities: at ventilatory threshold 1 (VT1), 15% below VT1, and 15% above VT1. The sessions were conducted with 48-h to 7-day intervals to ensure complete recovery. A single bout of exercise increased the circulating concentration of interleukin-6 (IL-6), which is secreted by contracting myocytes. This elevation triggered an anti-inflammatory cascade, thereby mitigating the excessive inflammatory response.

The findings demonstrated that, regardless of intensity, the levels of IL-6, IL-8, IL-10, and IL-4 increased in all participants. However, individuals with SCI exhibited higher levels of pro-inflammatory factors, including IL-1β, IL-2, and tumor necrosis factor-alpha (TNF-*α*), than able-bodied individuals, while displaying lower levels of anti-inflammatory factors such as IL-1ra, IL-4, and IL-10. Thus, it can be inferred that individuals with SCI require relatively intense HIET to counteract the progressive decline in the acute systemic anti-inflammatory cytokine response. Achieving a balance between pro-inflammatory and anti-inflammatory cytokine levels similar to that of the general population may alleviate excessive inflammatory responses.

### 2.4 Bone and skeletal muscle benefits

HIET has been demonstrated to alleviate cramping, enhance bone density, and improve myasthenia gravis more effectively than exercises of lower intensity. Paralysis following SCI frequently leads to neurogenic disuse osteoporosis, significantly increasing the risk of fractures in the distal femur and proximal tibia (46). Alterations in the excitability of supraspinal inhibitory pathways, combined with heightened motor neuron excitability after SCI, contribute to spasticity (47). Additionally, prolonged bed rest and diminished central nervous system control of skeletal muscles in individuals with SCI may cause muscle atrophy, attributed to changes in acetylcholine receptor subtypes and reduced acetylcholinesterase activity (39).

Chen et al. (48) (see Table 2 [4]) demonstrated that HIET accelerates systemic fluid circulation in growing rats while enhancing the metabolism and absorption of minerals and related substances, thereby promoting calcium and phosphorus ion deposition in bones, including the tibia, knee, and hip joints. Compared to low- and moderate-intensity endurance training, HIET yielded superior improvements in bone mineral density, bone structure, and bone tissue, as well as increased levels of osteocalcin, alkaline phosphatase, and anti-tartrate-resistant acid phosphatase during the growth period. These findings suggest that HIET may lower the risk of fractures in the distal femur and proximal tibia.

Gong (49) proposed that HIIT, a form of HIET, stimulates the potential of myocyte responses, promoting skeletal muscle hypertrophy more effectively than moderate-intensity continuous training. Sprint interval training, a subset of HIIT, was found to induce adaptive changes in rat adenylate-activated protein kinase (AMPK) through sprint interval exercise, enhancing the expression and translocation of glucose transporter 4 (GLUT4) in skeletal muscle and mitigating skeletal muscle cell damage caused by exhaustive exercise (50) (see Table 2 [9]).

Fang (51) (see Table 2 [5]) observed that HIET stimulated brain-derived neurotrophic factor (BDNF) and TrkB synthesis in SCI rats more effectively than low- and moderate-intensity training, ameliorating spasticity in the lower limbs of individuals with incomplete SCI. Similarly, Zhang et al. (47) (see Table 1 [7]) established a positive correlation between exercise intensity and spasticity improvement in individuals with SCI. Patients were categorized into three groups: conventional rehabilitation, low-intensity training, and high-intensity training. Both exercise groups utilized the MOTOmed intelligent exercise trainer to train lower limbs in conjunction with conventional rehabilitation. Low intensity was defined as 8–10 on Borg’s Perceived Exertion Scale (BPE), while high intensity was rated at 14–15. Spasticity in the ankle plantar flexor calf triceps was assessed using the Modified Ashworth Scale (MAS) and Modified Tardieu Scale (MTS). The results confirmed a positive correlation between training intensity and spasticity improvement.

Furthermore, high-intensity treadmill training has been shown to enhance neuromuscular synergy in individuals with SCI, thereby improving muscle coordination, increasing movement efficiency and accuracy, and facilitating motor function recovery (52) (see Table 1 [8]).

### 2.5 Sensory function benefits

Individuals with SCI often develop neuropathic pain, including abnormal pain, spontaneous pain, and nociceptive sensitization (53). Exercise training has been shown to mitigate neuropathic pain by strengthening sensory pathways, enhancing neuroplasticity, activating anti-inflammatory mechanisms, and suppressing inflammatory mediators and neurotransmitters involved in pain pathways (54). Exercise also modulates *γ*-aminobutyric acid levels in the dorsal horn of the spinal cord through TrkB signaling, alleviating mechanical allodynia and thermal hyperalgesia in rats with incomplete SCI (55).

Although few studies have explored the impact of exercise intensity on neuropathic pain, HIET is hypothesized to exert a more substantial influence on sensory pathways, neuroplasticity, and anti-inflammatory responses than low- or moderate-intensity exercise. Consequently, the potential of HIET in alleviating neuropathic pain warrants further investigation.

### 2.6 Psychology and daily life benefits

HIET has been shown to provide patients with SCI a heightened sense of security and control over their bodies, fostering hope and enabling them to achieve their goals (56). This approach has demonstrated efficiency in achieving desirable results within a short timeframe (57, 58) (see Table 1 [9, 10]), thereby reducing hospitalization costs and expediting the resumption of normal life activities. Training conducted on surfaces resembling those encountered in daily life, such as running tracks, has been found to facilitate reintegration into real-world activities more effectively (17).

### 2.7 Other benefits

SCI results in motor and sensory deficits as well as autonomic dysfunction. Hyporeflexia or hyperactivity of the urethral and sphincter muscles and dysfunction in urethral-sphincter synergy are typical symptoms of SCI-induced abnormal voiding. Gastrointestinal dyskinesia associated with SCI includes gastric dilatation, delayed gastric emptying, and reduced propulsive transport throughout the gastrointestinal tract (59). Interestingly, the functions of the urinary and digestive systems may be improved through enhanced neural stimulation induced by HIET, although the underlying mechanisms remain unclear (60, 61).

## 3 Potential adverse effects of HIET

**Figure 2:**
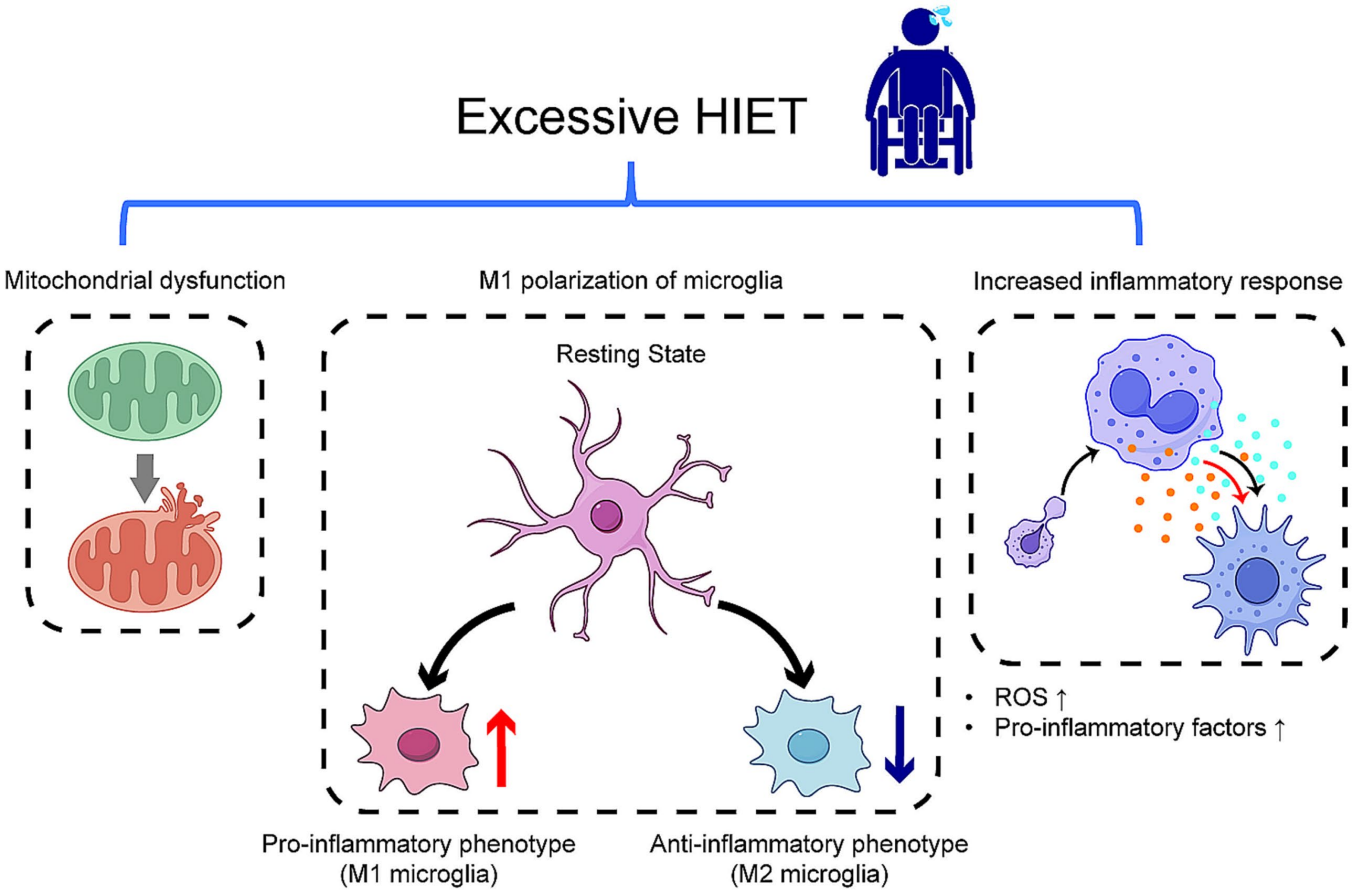
Effects of excessive HIET on spinal cord histopathological changes excessive exercise intensity can lead to heightened inflammation due to increased levels of pro-inflammatory or anti-inflammatory factors, resulting in diminished immunity, elevated ROS, mitochondrial swelling, and M1 polarization of microglia. These adverse effects hinder neuronal repair and contribute to secondary damage. ROS, reactive oxygen species.

Although HIET offers neuroprosthetic benefits for individuals with SCI, because of excessive exercise intensity, duration and frequency of practice, it also presents certain challenges (Figure 2), including the potential for excessive inflammatory responses, impaired mitochondrial function, all of which can exacerbate secondary injuries. Zhan et al. (62) (see Table 2 [6]) observed that SCI mice undergoing HIET exhibited reduced endurance during training and a higher mortality rate compared to mice subjected to low- or moderate-intensity training. Excessive HIET poses two principal risks: (1) when anti-inflammatory factors such as interleukin (IL)-10 and IL-4 predominate, the inflammatory response is suppressed excessively, leading to compromised immunity and increased susceptibility to infections such as urinary tract infections; (2) when pro-inflammatory factors such as IL-6 and IL-8 dominate, the inflammatory response intensifies, exacerbating secondary injuries (16) (see Table 1 [3]).

Furthermore, while HIET enhances mitochondrial function, it may also elevate ROS levels, aggravating local tissue inflammation and accelerating tissue damage (34, 63). Although HIET promotes brain-derived neurotrophic factor (BDNF) production, excessive BDNF levels may result in adverse neuronal plasticity due to insufficient neuromodulation mechanisms, potentially triggering M1 polarization of spinal microglia (64). This process can heighten nociceptive sensitivities, promote hyperexcitability, and strengthen neuronal circuits through activation of the TrkB signaling pathway, leading to persistent chronic pain. Elevated BDNF levels may also cause mitochondrial swelling and myocyte damage (42, 55, 64, 65) (see Table 2 [7]).

Remarkably, SCI may also impair reproductive function. A previous study reported that HIET decreases sperm quality in SCI rats (66) (see Table 2 [8]). However, the effects and mechanisms underlying SCI-related reproductive dysfunction require further investigation.

## 4 Research limitations

To date, few studies have evaluated the effects of HIET on functional recovery following SCI, and a standardized definition of HIET remains absent. Many studies have not adequately accounted for gender differences or the influence of other treatment modalities as part of a comprehensive SCI management regimen. Clinical studies often adopt conservative definitions of HIET for safety considerations, limiting the reliability and generalizability of the results. Although evidence suggests that HIET enhances functional recovery efficiency and effectiveness after SCI, it is not widely implemented to prevent secondary injuries due to the lack of precise evaluation criteria. Additionally, the absence of standardized intensity thresholds in animal models highlights the need for improved understanding and definition of “high intensity.”

### 4.1 Misconceptions about exercise intensity

Debates regarding the definition and safety of exercise intensity have hindered the adoption of HIET in the physical rehabilitation of patients with neurological injuries (17). Many patients with SCI have an incomplete and inadequate understanding of exercise intensity, often failing to distinguish between moderate and high intensity. Furthermore, miscommunication and cognitive discrepancies between healthcare professionals and patients can result in insufficient exercise intensity or the conflation of intensity with frequency and duration, thereby diminishing rehabilitation effectiveness (58) (see Table 1 [10]).

HIET is typically categorized into aerobic and resistance exercise. According to the American College of Sports Medicine Guidelines for Exercise Testing and Exercise Prescription (9th Edition) (67), exercise intensity is classified as follows: low intensity (<57% of maximum heart rate), lower intensity (57–<64%), moderate intensity (64–<76%), higher intensity (76–<96%), and HIET (96–100%). While this classification is broadly applicable, specific testing protocols and guidelines tailored to individuals with SCI are required to develop personalized treatment plans for optimal outcomes. Moreover, healthcare professionals must prioritize patient education, emphasizing the significance of exercise intensity and clearly explaining training methodologies, indicators, and metrics to enhance comprehension and adherence to rehabilitation protocols.

### 4.2 Flaws in monitoring exercise intensity

The commonly employed method for monitoring exercise intensity is heart rate measurement, which is practical for real-time monitoring during exercise (18, 68, 69) (see Table 1 [4, 11]). However, Fahey et al. (17) highlighted that individuals with SCI may exhibit reduced neuromuscular force due to lower extremity weakness, which limits their cardiovascular response. Consequently, the measured maximum heart rate may underestimate the actual value, leading to overestimation of exercise intensity responses. Additionally, the use of medications such as beta-blockers to manage comorbidities in individuals with SCI can further compromise the accuracy of heart rate measurements (70). Therefore, further research is essential to elucidate the effects of SCI and related medications on heart rate responses to facilitate the development of improved treatment regimens.

### 4.3 Physical and psychological limitations

SCI-induced physical dysfunction significantly impairs the ability to complete exercises and movements, leading to frustration, depression, and psychological fear among patients. Studies have demonstrated that HIET may decrease the willingness to train in SCI-affected rats due to the challenges in execution, thereby reducing training efficiency (71). Sterling et al. (56) reported that physical impairments resulting from SCI heighten patients’ fear of falling during exercise. Consequently, individuals must exert greater focus on seemingly simple movements, leading to elevated levels of fatigue.

Additionally, patients have indicated that exercise equipment often cannot adapt to the real-time changes in their physical condition. The absence of proprioception makes it difficult to maintain stability on dynamic platforms, causing discomfort and strain during treadmill use. In such conditions, patients are also required to monitor their heart rate to maintain exercise intensity, and the constant shift in attention negatively impacts the continuity of their exercise routines. Therefore, conducting exercise training in outdoor environments, when feasible, could facilitate patient adaptation to daily life and positively influence their mood (72, 73).

To enhance exercise intensity monitoring, patients should not rely solely on visual prompts displayed on screens. Sports headphones can be utilized to provide auditory cues for exercise intensity through voice prompts, allowing for dynamic adjustments. In cases where training must be conducted indoors, virtual reality (VR) technology may create a visually secure and stimulating environment, alleviating the monotony of training sessions (74). Furthermore, real-time pressure detection could be integrated to adjust the treadmill’s speed automatically, ensuring both exercise intensity and patient safety, thus alleviating psychological barriers.

### 4.4 Incomplete design of relevant studies

The proportion of female subjects in clinical trials related to SCI remains disproportionately low, and there is an absence of heart rate parameters tailored specifically to SCI individuals, resulting in imprecise outcomes. Additionally, animal studies related to HIET are limited in scope.

Although the incidence of SCI is slightly higher in males than females (75), many clinical trials on exercise training intensity in SCI populations exhibit an imbalanced sex ratio, with female participants being underrepresented (76). This disparity has significant implications, as female SCI patients may not receive appropriately tailored treatments and could face unnecessary risks.

Most studies on exercise prescription for SCI adopt a conservative approach to intensity. The criteria in many trials are derived from maximal heart rate data of able-bodied individuals, failing to account for variations in cardiovascular dynamics post-SCI (17). Such oversights introduce errors in clinical trials and undermine the efficacy of exercise interventions in the SCI population. Future clinical trials must ensure gender balance to generate specialized, accurate exercise intensity data.

Moreover, studies investigating the progression of exercise intensity in SCI animal models remain sparse. A summary of HIET research in animal models is provided in Table 2. Current animal protocols lack a standardized framework for exercise intensity progression, often relying on platform training with intensity achieved by controlling platform speed. Some experiments determine the maximal speed of mice and set training speed proportionately, while others directly set fixed speeds. However, general criteria for high intensity in laboratory or clinical settings overlook the physiological differences between able-bodied individuals and SCI patients, compromising the accuracy and validity of experimental data.

## 5 Recommendations for exercise programs

### 5.1 HIET program design

HIET has been shown to improve training efficiency, reduce recovery time, and activate protective physiological mechanisms (19). However, no single treatment modality is sufficient for SCI recovery. HIET should be integrated with complementary therapies, such as cellular therapy, laser acupuncture, functional electrical stimulation, and brain-computer interfaces, to enhance therapeutic outcomes (77).

HIET, when improperly implemented, can lead to additional injuries. High-intensity interval training (HIIT) has been found to be more effective than continuous exercise in mitigating muscle fiber damage caused by sustained exertion. HIIT also enhances AMP-activated protein kinase (AMPK) phosphorylation, leading to increased expression and translocation of glucose transporter protein 4 (GLUT4) in skeletal muscles (50) (see Table 2 [9]). Consequently, intermittent exercise improves exercise capacity more effectively than continuous exercise. Based on the findings of various studies, HIIT is considered a preferred modality for SCI rehabilitation.

HIET is also associated with regulation of ROS and inflammatory mediators in the body. HIIT protocols should begin with moderate or low-intensity exercises, progressively increasing intensity to facilitate adaptation in both animal models and humans. During HIET, patients should aim to maintain their heart rate between 70 and 80% of their maximum heart rate, a target critical for achieving the desired exercise intensity while ensuring safety. Also, given the effects of medications on heart rate, patients can combine heart rate and exertion scales to control exercise intensity. This adaptability in training design enhances patient confidence and optimizes program effectiveness.

The use of exoskeletons in HIET programs can reduce exercise intensity; thus, reliance on such devices should be minimized, or exercise intensity should be increased proportionally (78). Additionally, training programs should prioritize restoring patients’ original functions rather than compensating for deficits. The design of exercises should replicate real-life scenarios, promoting convenience and efficiency to expedite reintegration into society.

### 5.2 Patients’ enjoyment and autonomy

Exercise training is inherently monotonous and exhausting, making patient autonomy a crucial element of rehabilitation. Cooperation and initiative from patients are essential, especially in HIET, which demands high levels of motivation to maintain adherence and maximize training outcomes (56).

To improve patient engagement, VR and other somatosensory technologies may be employed to enhance the entertainment value of training. These tools can provide immersive, professionally guided movement experiences, even within home settings. Medical staff should also encourage patients to overcome psychological barriers.

Community-based rehabilitation plays a pivotal role in post-hospitalization training. Medical institutions should collaborate with community organizations to strengthen patient education. Communities must also be equipped with adequate personnel and resources to support rehabilitation programs effectively.

### 5.3 Advocacy for future research

In clinical research, the maximum heart rate values of individuals with SCI should be systematically investigated to refine exercise prescriptions. Structural changes in the body, pharmacological interventions, gender differences, and other relevant factors must be incorporated to ensure accurate monitoring during clinical trials. These considerations will optimize the intensity and modalities of exercise training, such as combining heart rate with perceived exertion scales, facilitating the clinical application of HIET for individuals with SCI. Furthermore, the integration of HIET with other therapeutic interventions could enhance the overall efficacy of SCI treatment.

In basic research related to SCI, the standardization of exercise intensity settings and progression protocols for animal experiments is essential. These protocols should align with the methodologies established for other disease models. Additionally, the effects and mechanisms of HIET on brain-derived neurotrophic factor (BDNF) merit focused investigation. Rather than merely promoting high levels of BDNF expression, it is critical to determine the optimal exercise intensity interval that achieves therapeutic benefits.

Moreover, the regulation of anti-inflammatory and pro-inflammatory factors during the inflammatory response induced by HIET warrants further study. This research could help minimize secondary injuries associated with SCI and create a favorable environment for spinal cord tissue repair.

## 6 Conclusion and limitations

HIET has the potential to promote the repair of spinal cord tissue structure and function, enhance cardiorespiratory performance, mitigate central nervous system degeneration, modulate inflammatory responses, and reduce systemic complications associated with SCI. The underlying mechanisms include increasing BDNF levels, promoting oligodendrocyte production, decreasing pro-inflammatory factors, elevating anti-inflammatory factors and regulatory T-cells (Tregs), and improving biomarkers of cardiometabolic risk. However, it is important to note that excessive exercise intensity can cause secondary injuries. Such adverse effects may result from elevated pro-inflammatory and anti-inflammatory factors, necessitating careful monitoring of the psychological and physical state of patients during training and developing individualized HIET plans and conduct further research to validate the benefits and address the risks. The present study has certain limitations. First, due to the paucity of existing research, this study does not differentiate between complete and incomplete SCI, which are distinct in clinical practice. Future research should address these distinctions, considering the varying implications of different spinal cord segments. Second, the limited number of studies on HIET in SCI has necessitated reliance on findings from CNS diseases unrelated to SCI for certain inferences and hypotheses in this article. Consequently, explicit and in-depth exploration of exercise training methodologies for SCI is an urgent priority for future research.

## References

[1] Liu P. Research Progress on treatment methods for spinal cord injury. J Mod Med Health. (2023) 39:1720–6. doi: 10.3969/j.issn.1009-5519.2023.10.022

[2] Collaborators GBDSCI. Global, regional, and National Burden of spinal cord injury, 1990-2019: a systematic analysis for the global burden of disease study 2019. Lancet Neurol. (2023) 22:1026–47. doi: 10.1016/S1474-4422(23)00287-9, PMID: 37863591 PMC10584692

[3] Eli I Lerner DP Ghogawala Z. Acute traumatic spinal cord injury. Neurol Clin. (2021) 39:471–88. doi: 10.1016/j.ncl.2021.02.004, PMID: 33896529

[4] Cao N Feng YP Xie JX. Interpretation of “clinical guidelines for Neurorestorative in spinal cord injury (2021 China version)”. Chin J Contemp Ncurol Neurosurg. (2022) 22:655–61. doi: 10.3969/j.issn.1672-6731.2022.08.002

[5] He L Chen JS Liu S Luo LY. The value of rehabilitation training for the recovery of neurological function and quality of life after spinal cord injury. Chinese Commun Phys. (2020) 36:173–4. doi: 10.3969/j.issn.1007-614x.2020.26.085

[6] Zhang FS Xie HX Gao DY Yang YH Di H Zhang J. Survey and analysis of rehabilitation training for people with spinal cord injury in Shanghai. Chin J Rehab Med. (2020) 35:1403. doi: 10.3969/j.issn.1001-1242.2020.11.014

[7] Xie QF Xie YY Hu Q Chen XL Lin JW Ying XW. Water treadmill training promotes the polar expression of aquaporin 4 and reduces tissue edema after spinal cord injury in rats. J Wenzhou Med Univ. (2023) 53:450–7. doi: 10.3969/j.issn.2095-9400.2023.06.003

[8] Zheng Y Mao YR Yuan TF Xu DS Cheng LM. Multimodal treatment for spinal cord injury: a sword of neuroregeneration upon neuromodulation. Neural Regen Res. (2020) 15:1437–50. doi: 10.4103/1673-5374.274332, PMID: 31997803 PMC7059565

[9] Wu J Li X Wang Q Wang S He W Wu Q. LncRNA/miRNA/mRNA ceRNA network analysis in spinal cord injury rat with physical exercise therapy. PeerJ. (2022) 10:e13783. doi: 10.7717/peerj.13783, PMID: 35923891 PMC9341448

[10] Chang F Zhang Q Xie H Yang Y Sun M Wu A. Effects of a rehabilitation program for individuals with chronic spinal cord injury in Shanghai, China. BMC Health Serv Res. (2020) 20:298. doi: 10.1186/s12913-020-05181-x, PMID: 32293434 PMC7158161

[11] Lucas-Osma AM Schmidt EKA Vavrek R Bennett DJ Fouad K Fenrich KK. Rehabilitative training improves skilled forelimb motor function after cervical unilateral contusion spinal cord injury in rats. Behav Brain Res. (2022) 422:113731. doi: 10.1016/j.bbr.2021.113731, PMID: 34979221

[12] Li NN Yang ZP Yang WW. Effects of Core muscle group combined with limb linkage rehabilitation training on key muscle strength and balance ability of patients with lumbar segmental incomplete spinal cord injury. Henan Med Res. (2020) 29:3734–6. doi: 10.3969/j.issn.1004-437X.2020.20.033

[13] Solinsky R Draghici A Hamner JW Goldstein R Taylor JA. High-intensity, whole-body exercise improves blood pressure control in individuals with spinal cord injury: a prospective randomized controlled trial. PLoS One. (2021) 16:e0247576. doi: 10.1371/journal.pone.0247576, PMID: 33661958 PMC7932070

[14] Afshari K Ozturk ED Yates B Picard G Taylor JA. Effect of hybrid Fes exercise on body composition during the sub-acute phase of spinal cord injury. PLoS One. (2022) 17:e0262864. doi: 10.1371/journal.pone.0262864, PMID: 35073366 PMC8786191

[15] Mahalakshmi B Maurya N Lee SD Bharath KV. Possible neuroprotective mechanisms of physical exercise in neurodegeneration. Int J Mol Sci. (2020) 21:5895. doi: 10.3390/ijms21165895, PMID: 32824367 PMC7460620

[16] Alves EDS Dos Santos RVT de Lira FS Almeida AA Edwards K Benvenutti M. Effects of intensity-matched exercise at different intensities on inflammatory responses in able-bodied and spinal cord injured individuals. J Spinal Cord Med. (2021) 44:920–30. doi: 10.1080/10790268.2020.1752976, PMID: 32298225 PMC8725751

[17] Fahey M Brazg G Henderson CE Plawecki A Lucas E Reisman DS. The value of high intensity locomotor training applied to patients with acute-onset neurologic injury. Arch Phys Med Rehabil. (2022) 103:S178–88. doi: 10.1016/j.apmr.2020.09.399, PMID: 33383032 PMC8581515

[18] Hutchinson MJ Goosey-Tolfrey VL. Rethinking aerobic exercise intensity prescription in adults with spinal cord injury: time to end the use of "moderate to vigorous" intensity? Spinal Cord. (2022) 60:484–90. doi: 10.1038/s41393-021-00733-2, PMID: 34880442 PMC9209328

[19] Jung KS Hutchinson MJ Chotiyarnwong C Kusumawardani MK Yoon SH Mikami Y. Dissonance in views between healthcare professionals and adults with a spinal cord injury with their understanding and interpretation of exercise intensity for exercise prescription. BMJ Open Sport Exerc Med. (2023) 9:e001487. doi: 10.1136/bmjsem-2022-001487, PMID: 36919123 PMC10008421

[20] Dolbow DR Davis GM Welsch M Gorgey AS. Benefits and interval training in individuals with spinal cord injury: a thematic review. J Spinal Cord Med. (2022) 45:327–38. doi: 10.1080/10790268.2021.2002020, PMID: 34855568 PMC9135438

[21] Huang CL Zhang Y Zhou YY Liu WW Zhao MM He FC. Effect of intensive rehabilitation nursing on cardiopulmonary function in patients with thoracolumbar spinal cord injury. Chin J Geriatr Care. (2020) 18:123–7. doi: 10.3969/j.issn.1672-2671.2020.03.049

[22] Astorino TA Hicks AL Bilzon JLJ. Viability of high intensity interval training in persons with spinal cord injury-a perspective review. Spinal Cord. (2021) 59:3–8. doi: 10.1038/s41393-020-0492-9, PMID: 32483336

[23] Farrow M Maher J Deere R Spellanzon B Williams S Thompson D. Effect of high-intensity interval training on cardiometabolic component risks in persons with paraplegia: results of a randomized controlled trial. Exp Physiol. (2024) 109:1253–66. doi: 10.1113/EP091803, PMID: 38924175 PMC11291867

[24] Hortobagyi T Vetrovsky T Balbim GM Sorte Silva NCB Manca A Deriu F. The impact of aerobic and resistance training intensity on markers of neuroplasticity in health and disease. Ageing Res Rev. (2022) 80:101698. doi: 10.1016/j.arr.2022.101698, PMID: 35853549

[25] Shi XH. Research Progress on the mechanism of sports training promoting the repair of spinal cord injury. Chongqing Med. (2021) 50:3389–94. doi: 10.3969/j.issn.1671-8348.2021.19.032

[26] Leech KA Hornby TG. High-intensity locomotor exercise increases brain-derived neurotrophic factor in individuals with incomplete spinal cord injury. J Neurotrauma. (2017) 34:1240–8. doi: 10.1089/neu.2016.4532, PMID: 27526567 PMC5359683

[27] Kiss K Bacova M Kisucka A Gálik J Ileninova M Kuruc T. Impact of endurance training on regeneration of axons, glial cells, and inhibitory neurons after spinal cord injury: a link between functional outcome and regeneration potential within the lesion site and in adjacent spinal cord tissue. Int J Mol Sci. (2023) 24:616. doi: 10.3390/ijms24108616, PMID: 37239968 PMC10218115

[28] An LB Liu CH Zhao BL Zhou XH Li WT. Effects of body weight support treadmill training with different exercise intensities on expressions of Trkb and Bdnf proteins in spinal cord tissue and its promotion effect on motor function recovery of rats with spinal cord injury. J Jilin Univ. (2019) 45:1389–94. doi: 10.13481/j.1671-587x.20190633

[29] Zhan ZX. Study of effect of treadmill exercise on Mtor pathway and motor function recovery in mice with spinal cord injury [master's thesis]. Chongqing: Chong Qing Medical University (2022).

[30] Anjum A Yazid MD Fauzi M Idris J Ng AMH Selvi A. Spinal cord injury: pathophysiology, multimolecular interactions, and underlying recovery mechanisms. Int J Mol Sci. (2020) 21:7533. doi: 10.3390/ijms21207533, PMID: 33066029 PMC7589539

[31] Zhou HY Cui F Shuang WB. The role and mechanism of mitochondrial dysfunction in progression of spinal cord injury. China Med Eng. (2020) 28:33–8. doi: 10.19338/j.issn.1672-2019.2020.09.009

[32] Zhou L. *Salvia Divinorum* attenuates the inflammatory damage of Β-amyloid in ad brain and its mechanism [Master's thesis]. Hunan: Central South University (2014).

[33] Zhang ZY Kang WM Zhang S Bo H. High-intensity interval training-induced neuroprotection of Hippocampus in app/Ps1 transgenic mice via upregulation of mitophagy. Chin J Rehab Med. (2020) 35:670–5. doi: 10.3969/j.issn.1001-1242.2020.06.005

[34] Gao ZRD. Research Progress of mechanism and monitoring of neuromuscular function remodeling and regeneration in sprint training. Bullet Sport Sci Technol. (2023) 31:219–23,65. doi: 10.19379/j.cnki.issn.1005-0256.2023.03.061

[35] Liu XX Zhou MW. Structural changes and functional remodeling of the brain after spinal cord injury. Chin J Rehab Med. (2021) 36:1026–30. doi: 10.3969/j.issn.1001-1242.2021.08.025

[36] Zhao C Bao SS Xu M Rao JS. Importance of brain alterations in spinal cord injury. Sci Prog. (2021) 104:368504211031117. doi: 10.1177/00368504211031117, PMID: 34242109 PMC10450736

[37] Li Y Cao T Ritzel RM He J Faden AI Wu J. Dementia, depression, and associated brain inflammatory mechanisms after spinal cord injury. Cells. (2020) 9:1420. doi: 10.3390/cells9061420, PMID: 32521597 PMC7349379

[38] Li Y Ritzel RM Khan N Cao T He J Lei Z. Delayed microglial depletion after spinal cord injury reduces chronic inflammation and neurodegeneration in the brain and improves neurological recovery in male mice. Theranostics. (2020) 10:11376–403. doi: 10.7150/thno.49199, PMID: 33052221 PMC7545988

[39] Sun P Ye XM Tian L Li JB Cheng RD. Effect of spinal cord injury on the expression of C-Fos in hypothalamus and neuronal apoptosis. China Modern Doctor. (2020) 58:45–8.

[40] He LW Guo XJ Zhao C Rao JS. Rehabilitation training after spinal cord injury affects brain structure and function: from mechanisms to methods. Biomedicines. (2023) 12:10041. doi: 10.3390/biomedicines12010041, PMID: 38255148 PMC10813763

[41] Redondo-Castro E Navarro X. Peripheral nerve alterations after spinal cord injury in the adult rat. Spinal Cord. (2013) 51:630–3. doi: 10.1038/sc.2013.57, PMID: 23774128

[42] Sabatier MJ Redmon N Schwartz G English AW. Treadmill training promotes axon regeneration in injured peripheral nerves. Exp Neurol. (2008) 211:489–93. doi: 10.1016/j.expneurol.2008.02.013, PMID: 18420199 PMC2584779

[43] Brennan FH Li Y Wang C Ma A Guo Q Li Y. Microglia coordinate cellular interactions during spinal cord repair in mice. Nat Commun. (2022) 13:4096. doi: 10.1038/s41467-022-31797-0, PMID: 35835751 PMC9283484

[44] Tian TaL X. Problems and challenges in regeneration and repair of spinal cord injury. Chinese J Tissue Eng Res. (2021) 25:3039–48.

[45] Walsh CM Gull K Dooley D. Motor rehabilitation as a therapeutic tool for spinal cord injury: new perspectives in immunomodulation. Cytokine Growth Factor Rev. (2023) 69:80–9. doi: 10.1016/j.cytogfr.2022.08.005, PMID: 36114092

[46] Sutor TW Kura J Mattingly AJ Otzel DM Yarrow JF. The effects of exercise and activity-based physical therapy on bone after spinal cord injury. Int J Mol Sci. (2022) 23:608. doi: 10.3390/ijms23020608, PMID: 35054791 PMC8775843

[47] Zhang JL Sou L Li XZ Yin JJ Wu QF Wang HX. Effects of different intensities of exercises on spasticity and concentration of serum brain-derived neurotrophic factor in patients with incomplete spinal cord injury. Chin J Rehab Med. (2022) 37:80–4. doi: 10.3870/zgkf.2022.02.003

[48] Chen ZG Ding HL Li L Wang C. Changes of bone metabolism after different intensity endurance exercises in growing rats. Chin J Tissue Eng Res. (2020) 24:3382–5588. doi: 10.3969/j.issn.2095-4344.2918

[49] Gong XW. Study on whether high-intensity interval training can promote skeletal muscle anabolism. Contemp Sports Technol. (2022) 12:1–4. doi: 10.16655/j.cnki.2095-2813.2201-1579-9022

[50] Zhang LM Liu JJ Lin XY Liu L Lu J. Mechanism by which high-intensity intermittent exercise improves skeletal muscle injury and enhances exercise capacity in rats. Chin J Tissue Eng Res. (2023) 27:5603–9. doi: 10.12307/2023.890

[51] Fang L. Research on the effect of exercise training on spasticity and its mechanism in rats after spinal cord injury [Master’s thesis]. Nanjing: Nanjing Medical University (2018).

[52] Ardestani MM Henderson CE Salehi SH Mahtani GB Schmit BD Hornby TG. Kinematic and neuromuscular adaptations in incomplete spinal cord injury after high- versus low-intensity locomotor training. J Neurotrauma. (2019) 36:2036–44. doi: 10.1089/neu.2018.5900, PMID: 30362878 PMC6599383

[53] Chen L Lei J Yu HJ. Progress of research on the mechanism and treatment of pathologic pain after spinal cord injury. Chin J Pain Med. (2022) 28:843–8. doi: 10.3969/j.issn.1006-9852.2022.11.008

[54] Palandi J Bobinski F de Oliveira GM Ilha J. Neuropathic pain after spinal cord injury and physical exercise in animal models: a systematic review and meta-analysis. Neurosci Biobehav Rev. (2020) 108:781–95. doi: 10.1016/j.neubiorev.2019.12.016, PMID: 31837360

[55] Li X Wang Q Ding J Wang S Dong C Wu Q. Exercise training modulates glutamic acid Decarboxylase-65/67 expression through TRKB signaling to ameliorate neuropathic pain in rats with spinal cord injury. Mol Pain. (2020) 16:1744806920924511. doi: 10.1177/1744806920924511, PMID: 32418502 PMC7235678

[56] Sterling MK Wouda MF Lahelle AF. A qualitative interview study on how people with incomplete spinal cord injury experience high-intensity walking exercise. Spinal Cord Ser Cases. (2021) 7:92. doi: 10.1038/s41394-021-00456-9, PMID: 34611134 PMC8492776

[57] Graham K Yarar-Fisher C Li J McCully KM Rimmer JH Powell D. Effects of high-intensity interval training versus moderate-intensity training on cardiometabolic health markers in individuals with spinal cord injury: a pilot study. Top Spinal Cord Inj Rehabil. (2019) 25:248–59. doi: 10.1310/sci19-00042, PMID: 31548792 PMC6743747

[58] McLeod JC Diana H Hicks AL. Sprint interval training versus moderate-intensity continuous training during inpatient rehabilitation after spinal cord injury: a randomized trial. Spinal Cord. (2020) 58:106–15. doi: 10.1038/s41393-019-0345-6, PMID: 31462757

[59] Hou S Rabchevsky AG. Autonomic consequences of spinal cord injury. Compr Physiol. (2014) 4:1419–53. doi: 10.1002/cphy.c130045, PMID: 25428850

[60] Hubscher CH Montgomery LR Fell JD Armstrong JE Poudyal P Herrity AN. Effects of exercise training on urinary tract function after spinal cord injury. Am J Physiol Renal Physiol. (2016) 310:F1258–68. doi: 10.1152/ajprenal.00557.2015, PMID: 26984956 PMC4935767

[61] Ouyang S Wang X Chen Y Deng L Yang X Hu S. Swimming training combined with fecal microbial transplantation protects motor functions in rats with spinal cord injury by improving the intestinal system. Neurosci Lett. (2023) 799:137104. doi: 10.1016/j.neulet.2023.137104, PMID: 36758789

[62] Zhan Z Pan L Zhu Y Wang Y Zhao Q Liu Y. Moderate-intensity treadmill exercise promotes Mtor-dependent motor cortical neurotrophic factor expression and functional recovery in a murine model of crush spinal cord injury (Sci). Mol Neurobiol. (2023) 60:960–78. doi: 10.1007/s12035-022-03117-6, PMID: 36385234

[63] Zhu ZH Zou HJ Song ZW Liu JB. Cellular microenvironment in nerve repair after spinal cord injury. Chin J Tissue Eng Res. (2023) 27:114–20. doi: 10.12307/2022.971

[64] Bai J Geng B Wang X Wang S Yi Q Tang Y. Exercise facilitates the M1-to-M2 polarization of microglia by enhancing autophagy via the Bdnf/Akt/Mtor pathway in neuropathic pain. Pain Physician. (2022) 25:E1137–51. PMID: 36288601

[65] Zhang X Yu B Cai BT. Effects of exercise intensities on expression of hypoxia inducible factor-1α Mrna in the skeletal muscle of rats. Chin J Orthop Trauma. (2006) 8:453–7. doi: 10.3760/cma.j.issn.1671-7600.2006.05.014

[66] Zhou XH. Study on the effect of weight-loss walking training on sperm quality in male rats with spinal cord injury [Master’s thesis]. Jilin: Jilin University (2020).

[67] ACSM. Acsm’s guidelines for exercise testing and prescription. 9th ed. Beijing: Beijing Sport University Press (2015).

[68] McMillan DW Maher JL Jacobs KA Nash MS Bilzon JLJ. Physiological responses to moderate intensity continuous and high-intensity interval exercise in persons with paraplegia. Spinal Cord. (2021) 59:26–33. doi: 10.1038/s41393-020-0520-9, PMID: 32681118

[69] Goosey-Tolfrey VL Hutchinson M Sharpe L. Infographic. Field-based methods for assessing exercise intensity in adults with spinal cord injury. Br J Sports Med. (2023) 57:203–4. doi: 10.1136/bjsports-2022-106226, PMID: 36450438

[70] Tsai SW Huang YH Chen YW Ting CT. Influence of Β-blockers on heart rate recovery and rating of perceived exertion when determining training intensity for cardiac rehabilitation. J Chin Med Assoc. (2015) 78:520–5. doi: 10.1016/j.jcma.2015.05.009, PMID: 26155925

[71] Fenrich KK Hallworth BW Vavrek R Raposo PJF Misiaszek JE Bennett DJ. Self-directed rehabilitation training intensity thresholds for efficient recovery of skilled forelimb function in rats with cervical spinal cord injury. Exp Neurol. (2021) 339:113543. doi: 10.1016/j.expneurol.2020.113543, PMID: 33290776

[72] Jannings W Pryor J. The experiences and needs of persons with spinal cord injury who can walk. Disabil Rehabil. (2012) 34:1820–6. doi: 10.3109/09638288.2012.665126, PMID: 22423597

[73] Jordan MM Berkowitz D Hannold E Velozo CA Behrman AL. Thinking through every step: how people with spinal cord injuries relearn to walk. Qual Health Res. (2013) 23:1027–41. doi: 10.1177/1049732313494119, PMID: 23774628

[74] Maggio MG Bonanno M Manuli A Onesta MP de R Quartarone A. Do individuals with spinal cord injury benefit from semi-immersive virtual reality cognitive training? Preliminary results from an exploratory study on an underestimated problem. Brain Sci. (2023) 13:945. doi: 10.3390/brainsci13060945, PMID: 37371423 PMC10296140

[75] Ding W Hu S Wang P Kang H Peng R Dong Y. Spinal cord injury: the global incidence, prevalence, and disability from the global burden of disease study 2019. Spine. (2022) 47:1532–40. doi: 10.1097/BRS.0000000000004417, PMID: 35857624 PMC9554757

[76] Liu J Gao H Li JJ. Epidemiology of patients with traumatic spinal cord injury and study on the influencing factors of hospitalization costs. Chin J Rehab. (2020) 35:139–42. doi: 10.3870/zgkf.2020.03.006

[77] Neurorestoratology IAoNaCAo. Interpretation of clinical Neurorestorative therapeutic guidelines for spinal cord injury (Ianr/Canr version 2019). Med J West China. (2020) 32:790–802. doi: 10.3969/j.issn.1672-3511.2020.06.003 PMC6939117 31908929

[78] Poggensee KL Collins SH. How adaptation, training, and customization contribute to benefits from exoskeleton assistance. Sci Robot. (2021) 6:eabf1078. doi: 10.1126/scirobotics.abf1078, PMID: 34586837

